# Supplementary material for: Ambitions and realities: Are Global Fund investments designed to achieve resilient and sustainable systems for health? Findings from the Global Fund Prospective Country Evaluation
Source: PLOS Glob Public Health. 2024 Nov 14;4(11):e0003914. doi: 10.1371/journal.pgph.0003914 (PMC11563383; doi:10.1371/journal.pgph.0003914)
Supplement: S1 Text — (DOCX) [file pgph.0003914.s001.docx]

# PCE 2020 Protocol on Operationalizing the 2S Framework (v1.0 July 14, 2020)

## Purpose of this document

This document provides guidance for how to analyze whether “direct” RSSH investments in current and upcoming Global Fund grants constitute health systems support or health systems strengthening This guidance builds from references to 4S in both the [RSSH](https://drive.google.com/drive/folders/1FXIp9D3BUm3Jc5vfi8tvIVKp0ONKBipp) and [sustainability](https://docs.google.com/document/d/1PoiBlvm3Sf0g1LEo9Ba4upit86p8UQeC/edit) guidance documents. Given the need for consistency in applying the framework to budgets in the 2017 and 2020 funding requests and across the four countries, GEP and CEP will work together closely on the coding process. By examining the change over time, we will assess whether countries have increased the proportion or Global Fund RSSH investments contributing to health systems strengthening (versus support).

## Health system investments: supporting vs. strengthening

The [TRP report on RSSH](https://www.theglobalfund.org/media/8093/trp_rssh2017-2019fundingcycle_report_en.pdf) investments in the 2017-2019 funding cycle applied the 4S framework (start-up, support, strengthening, sustainability), which was an expansion of the framework proposed by [Grace Chee et al. (2013)](https://onlinelibrary.wiley.com/doi/full/10.1002/hpm.2122) describing systems support vs. systems strengthening (hereafter “2S”) as visualized through the health systems cube (Figure 1). Through the TRP’s review, it was noted that the ‘start-up’ and ‘sustainability’ categories of 4S (the outer two S) were not applied very often, meaning that most Global Fund investments fell into either the ‘support’ or ‘strengthening’ categories (the middle two S) within the 4S continuum. For that reason, and in consultation with the TRP, the PCE will apply the 2S framework, which will also help to increase coding consistency by having fewer categories.

**Figure 1**. Systems Support and Systems Strengthening: Health Systems Cube (Chee et al. 2013).

**
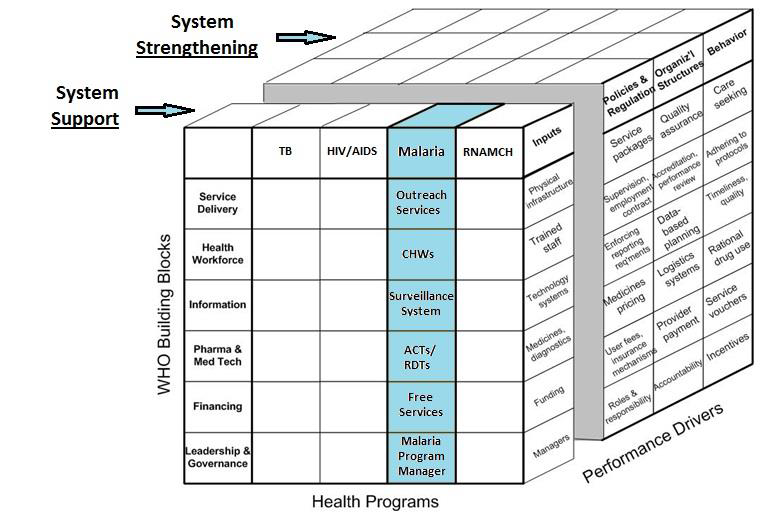
**

The health system cube Y axis includes the *WHO building blocks*, which are considered key functions of the health system. The X axis includes illustrative *disease‐specific programs* that deliver critical services, while the third dimension along the Z axis includes *performance drivers*, including inputs (systems support), policies, and regulations, organizational structures, and behaviors (systems strengthening) (Chee et al. 2013). Our analysis will **assess whether proposed RSSH activities contribute to systems support versus system strengthening** in line with Figure 1 above. Per Chee et al. (2013):

- “Supporting the health system can include any activity that improves services, from distributing mosquito nets to procuring medicines. These activities improve outcomes primarily by increasing inputs.”
- “Strengthening the health system is accomplished by more comprehensive changes to performance drivers such as policies and regulations, organizational structures, and relationships across the health system to motivate changes in behavior and/or allow more effective use of resources to improve multiple health services.”

The TRP methodology notes that funding requests examples that were health systems support oriented included requests for cars, computers, phones, travel costs for routine monitoring, furniture and office equipment, payments for fuels and maintenance of vehicles, cost for regular training or overseas training, software, reimbursement for importation, among others. Whereas funding requests characterized by more health systems strengthening interventions included requests for upscaling of volunteer networks; developing protocols for data quality monitoring; developing standard operating procedures for quality control in laboratories; transferring of the procurement system of Global Fund into the national procurement systems; digitizing HMIS data; developing strategies to engage with the private sector; providing technical assistance for DHIS2 roll-out, improving procurement and supply chain procedures including e-LMIS, and establishing medicine regulatory authority, among others.

## Data Sources for RSSH interventions/activities

PCE will rely on up to three budget types as source documents for applying 2S, pending data availability:

1. Final approved budgets following grant making (2017)
   - To be independently coded by two GEP and compared to review any inconsistencies and reach consensus on 2S codes
   - The final approved grant making budget is preferable to the funding request budget, as it represents the RSSH allocation actually planned for implementation.
2. Funding request budgets submitted to TRP (2020)
   - To be independently coded by GEP and CEP (and then compared in small working group to review inconsistencies in coding and to reach consensus on 2S codes)
3. Final approved budgets following grant making (2020) for HIV in GTM
   - This may not be available for all countries by the end of 2020. Where available following grant making, to be independently coded by GEP and CEP (and then compared to review inconsistencies in coding and to reach consensus on 2S codes)

In addition to the budget data, the funding request narrative description of RSSH investments should be used as a secondary source for triangulating with budget information when examining interventions (or activities) and applying the 2S framework. The funding request narrative is often not well aligned with the budget, unfortunately, but can still often be helpful in understanding the overall scope of the RSSH interventions.

## Steps for operationalizing 2S application to RSSH activities in Global Fund grants

4.1 Review the RSSH budget data

Relevant budget data will be extracted by GEP for each country to ensure we are using a systematic data format across countries (see [2S Analysis Template](https://docs.google.com/spreadsheets/d/1vV8PoxH5-ofOPaSWnaaYpLMLfeaQ--_jmDQO5iGpjow/edit#gid=1980613073)). In line with the detailed budgets, the template includes columns (shaded green) for the extracted data: Grant, Grant Period, Module, Intervention, Activity Description, Cost Input, and Budget. Additional columns (shaded blue or purple) for data entry by coders include Scope, Longevity, Approach, Designation (Supporting vs. Strengthening) and Justification.

Review budget data for each line item, examining the module, intervention, and activity description. In addition, review the Cost Input categorization. Together, these four data elements should yield sufficient information to understand the RSSH activity. In some cases, where this data is insufficient, the funding request narrative can be referred to for additional description of the RSSH interventions.

| **HMIS/M&E Investments**  RSSH investments in many PCE countries contain substantial investments in HMIS/M&E modules. While as for other modules, determining whether HMIS/M&E investments are strengthening or supporting requires careful review of the activity description, our review of the 2017 budgets has revealed a few patterns. Generally speaking, activities (meetings, etc) related to data validation (especially when part of the analysis/review intervention category) can be considered strengthening. Activities related to monitoring the performance of the Global Fund grants themselves (as opposed to strengthening country M&E systems) should be considered supporting. Some activities aimed at improving internet connectivity to support information systems strengthening can also be considered strengthening because, although an input, efforts to improve the collection and use of data is persistently hampered by poor connectivity, and therefore this may be a foundational investment upon which broader strengthening efforts are dependent. Other activities (training, supervision, etc) related to the roll-out of DHIS2 (or similar system) can be considered strengthening. |
| --- |

### 4.2 Consider the Scope, Longevity, and Approach of each RSSH intervention/activity pair, and the cost input category

Drawing from the TRP’s methodology, we will apply three criteria--scope, longevity, and approach--to assess each RSSH intervention/activity pair in the budget. These three criteria, along with the Cost Input categorization, will be taken together in determining the designation of “supporting” or “strengthening”. With the exception of Justification (which requires the coder to type a justification for why they selected supporting or strengthening, these columns all contain dropdown menus for the definitions of Scope, Longevity, Approach, and Designation to ease the coding process. The criteria for a system strengthening intervention include:

- **Scope**: activities have impact across health services and outcomes
- **Longevity**: effects will continue after activities end
- **Approach**: revise policies and institutional relationships to change behaviors and resource use to address identified constraints in a more sustainable manner

The definitions of Scope, Longevity, and Approach included in the table below will be used for determining whether an activity is systems support versus systems strengthening.

| **Parameter** | **System Support** | **System Strengthening** |
| --- | --- | --- |
| **Scope** | May be focused on a single disease or intervention | Activities have impact across health services and outcomes; and systems may be integrated into the overall health sector |
| **Longevity** | Effects limited to period of funding | Effects will continue after funded activities end |
| **Approach** | Provide inputs to address identified system gaps | Revise policies and institutional relationships to change behaviors and resource use to address identified constraints in a more sustainable manner |

In many cases, if the majority of the three (scope, longevity and approach) criteria are designated support or strengthening (i.e. at least 2 out of 3), that will be sufficient to determine the final designation for the row. There are some cases, where one or more of the criteria may be designated as unclear, even after considering the cost input category (see below), resulting in a 1-1 tie. These instances will require more of a qualitative judgment to arrive at a final determination and should be discussed between coders to ensure agreement.

**Cost input categories**

There are minimum budget requirements for Global Fund funding request submissions, including modules and their related interventions (selected from prescribed list in the Modular Framework Handbook) and cost groupings and cost inputs selected from a prescribed list (see Appendix 1, p. 77 of [Global Fund guidelines for budgeting](https://www.theglobalfund.org/media/3261/core_budgetinginglobalfundgrants_guideline_en.pdf)). In addition to considering the scope, longevity and approach criterion in determining whether an activity is supporting or strengthening, certain cost categories (as indicated in the Table below) can inform this categorization.

| **Cost Grouping** | **Cost Input Categories** | **Supporting / Strengthening** |
| --- | --- | --- |
| 1. Human resources | 1.1 Salaries - program management  1.2 Salaries -outreach workers, medical staff and other service providers  1.3 Performance-based supplements, incentives  1.4 Other human resources costs  1.5 Severance costs *(added to 2019 guidelines)* | 1.1/1.2 Considered ***supporting*** as a health system input--including payment for salaries and other financial incentives  1.3 Performance-based supplements and incentives are the exception to this rule and can be considered ***strengthening***. |
| 2. Travel-related costs | 2.1 Training-related per diems/transport/other costs  2.2 TA-related per diems/transport/other costs  2.3 Supervision/surveys/data collection-related per diems/transport/other costs  2.4 Meeting/advocacy-related per diems/transport/other costs  2.5 Other transportation costs | Most often considered ***supporting*** as a health system input.  2.1 Training-related per-diems/transport/other costs should be considered ***supporting***, unless activity description clearly describes strengthening capacity in relation to data use, data validation meetings, financial management, PSM, M&E, or other systems strengthening related area)  2.4 Meeting related costs generally considered ***supporting*** unless the activity description indicates that the purpose is for policy change or development. |
| 3. External professional services | 3.1 Technical assistance fees/consultancy fees  3.2 Fiscal/fiduciary agent fees  3.3 External audit fees  3.4 Other external professional services  3.5 Insurance related costs | 3.1 Technical assistance may be considered ***strengthening*** but this determination depends upon review of the activity description. |
| 4. Health products -- pharmaceutical products | 4.1 Antiretroviral medicines  4.2 Anti-tuberculosis medicines  4.3 Antimalarial medicines  4.4 Opioid substitutes medicines  4.5 Opportunistic infections and STI medicines  4.6 Private sector subsidies for ACTs (co-payment to 4.3)  4.7 Other medicines | Most often considered ***supporting*** as a health system input (e.g. medicines for HIV, TB, malaria); *unlikely to be tagged within RSSH modules* |
| 5. Health products -- non-pharmaceuticals | 5.1 Insecticide-treated nets (long-lasting insecticidal nets/insecticide-treated nets)  5.2 Condoms –male  5.3 Condoms –female  5.4 Rapid diagnostic tests  5.5 Insecticides  5.6 Laboratory reagents  5.7 Syringes and needles  5.8 Other consumables  5.9 Private sector subsidies for rapid diagnostic tests (Co-payments to 5.4) | Most often considered ***supporting*** as a health system input (e.g. bednets, condoms, RDTs, insecticides, reagents, syringes); *unlikely to be tagged within RSSH modules* |
| 6. Health products -- equipment | 6.1 CD4 analyzer/accessories  6.2 HIV viral load analyzer/accessories  6.3 Microscopes  6.4 TB molecular test equipment  6.5 Maintenance and service costs for health equipment  6.6 Other health equipment | *Unlikely to be tagged within RSSH modules* |
| 7. Procurement and supply chain management costs | 7.1 Procurement agent and handling fees  7.2 Freight and insurance costs (health products)  7.3 Warehouse and storage costs  7.4 In-country distribution costs  7.5 Quality assurance and quality control costs  7.6 Procurement and supply management customs duties and clearance charges  7.7 Other procurement and supply management costs | Cost inputs in this group are most often considered ***supporting***  7.5 (quality assurance and quality control costs) and 7.7 (other procurement and supply management costs) are possible exceptions which might be considered ***strengthening***, depending on the activity description |
| 8. Infrastructure | 8.1 Furniture  8.2 Renovation/constructions  8.3 Infrastructure maintenance and other infrastructure costs |  |
| 9. Non-health equipment | 9.1 IT -computers, computer equipment, software and applications  9.2 Vehicles  9.3 Other non-health equipment  9.4 Maintenance and service costs non-health equipment | 9.1 IT equipment can typically be categorized as ***strengthening*** if it is intended to build data systems capacity. Equipment to facilitate stand-alone disease-specific activities may be considered support, but we haven’t seen examples of this yet.  9.2 Vehicles are always ***support***.  9.3 Varies, so closely review activity descriptions. Lab-strengthening equipment purchases are generally considered to be strengthening, and tend to fall within this cost input category |
| 10. Communication material and publications | 10.1 Printed materials (forms, books, guidelines, brochure, leaflets, etc.)  10.2 Television/radio spots and programs  10.3 Promotional material (t-shirts, mugs, pins, etc.) and other communication material and publications costs | All inputs within this cost grouping are generally considered to be ***supporting*** |
| 11. Indirect and overhead costs | 11.1 Office-related costs  11.2 Unrecoverable taxes and duties  11.3 Indirect cost recovery -% based  11.4 Other PA costs  11.5 Shared costs | All inputs within this cost grouping are generally considered to be ***supporting*** |
| 12. Living support to client/target population | 12.1 Support to orphans and other vulnerable children (school fees, uniforms, books, etc.)  12.2 Food and care packages  12.3 Cash incentives/transfer to patients/beneficiaries/counselors/ mediators  12.4 Microloans and microgrants  12.5 Other costs related to living support to client/target population | *Unlikely to be tagged within RSSH modules* |
| 13. Payment for results | *Added to 2019 guidelines:*  13.1 Results Based Financing  13.2 Activity Based Contracts, Community Based Organizations and other service providers  13.3 Incentives for Principal Recipient and Sub-Recipients staff members  13.4 Incentives for Community Health Workers (CHW), outreach workers, medical staff and other service providers | 13.1 and 13.4 are generally considered to be considered ***strengthening*** |

4.3 Review the funding request narrative to inform categorization

In some cases, the intervention/activity description and cost-inputs are very vague and provide insufficient information on which categorize activities as supporting or strengthening. In these instances, before making a final determination refer back to the funding request narrative to see if there is additional context around interventions to help inform the final categorization. If no such information exists, then apply the ‘unclear’ category. *Note: In our review of the 2017 budgets, we found very few interventions which required us to return to the funding request narratives, and that could not be categorized after exhausting all of these options*.

4.4 Review the other coder’s categorization and identify any discrepancies

Each budget will be coded twice, by one CEP and one GEP member. After completing coding of the budgets, assign one person to compare and identify any lines where the final designation of supporting vs. strengthening is not consistent between coders. Schedule a call to discuss each of those discrepancies and align on the final designation. If needed, consult with other members of the 2S working group to see if parallels can be drawn with other countries.

4.5 Quantify the proportion of the RSSH funds allocated to supporting vs. strengthening investments.

When final designations have been determined, quantify the proportion of funds going to supporting vs strengthening investments. This can then be compared to 2017 investments to see whether there is evidence of increased allocation of funds toward strengthening (which could be considered evidence of a ‘changing trajectory’, or whether the allocations are similar to 2017 (which could be considered evidence of ‘business as usual’). Depending on what we find, we may drill down to look in greater detail at change for specific modules or intervention categories (which may be related to focus topics, e.g. HMIS/M&E) and may consider using Tableau to help illustrate some of these findings. We will continue to build out our approach to this as we explore the data and patterns or findings begin to emerge.
