# Supplementary material for: Ambitions and realities: Are Global Fund investments designed to achieve resilient and sustainable systems for health? Findings from the Global Fund Prospective Country Evaluation
Source: PLOS Glob Public Health. 2024 Nov 14;4(11):e0003914. doi: 10.1371/journal.pgph.0003914 (PMC11563383; doi:10.1371/journal.pgph.0003914)
Supplement: S2 Text — (DOCX) [file pgph.0003914.s002.docx]

**KII questions for PCE extension 2021**

**1. RSSH:** This section includes three main components, including understanding:

1. Where Global Fund RSSH investments fit within the wider landscape, including comparative advantage and added value relative to domestic or other donor support.
2. The use/understanding of ‘system support’ vs. ‘system strengthening’ by the CCM and government stakeholders, including how decisions are made around RSSH investments and how prioritization of RSSH could be conducted/improved.
3. RSSH performance monitoring and low uptake of RSSH modular framework indicators

*Target stakeholders:*

- Health systems experts involved in NFM3 FR/GM -- from government and partners
- CCM members with health systems expertise
- Others?

| **(a) Global Fund RSSH investments in the wider funding landscape**: | |
| --- | --- |
| **Guiding Questions** | **Probes** |
| How do Global Fund investments in RSSH align to the investments of government and other donors/funders in health systems strengthening? | - Are Global Fund investments in similar areas to those of other donors *complimentary*, and designed to leverage the investments of others? - Is the Global Fund sufficiently engaged either itself or through its partners in national health sector planning and financing discussions? |
| How does the Global Fund’s investment in health systems strengthening add value to the investments made by others? | - What activities are funded by the Global Fund that would not be supported by others? - Is added value derived from RSSH investments having an explicit focus on the three diseases? - Do Global Fund RSSH investments have a ‘catalytic’ effect in supporting (a) the implementation of Global Fund grants; and (b) broader health service delivery? If so, please describe how? |
| **(b) Support vs. Strengthening and RSSH investment design/prioritization:**  In line with our prior analysis, the Global Fund makes a distinction between investments in RSSH systems support - i.e. those ‘that focus on short-term, input-focused support (such as vehicles, travel, training costs, equipment and others)’ and systems strengthening - i.e. that build capacity to lead to sustainable results (such as through strengthening management, improving accountability mechanisms, and empowering service providers).  Despite Global Fund guidance suggesting that RSSH investments should ‘shift’ from systems support to systems strengthening, the synthesis report highlighted that RSSH investments in all PCE countries are mostly for systems support. However, the evidence on how and why these decisions are taken is weak. | |
| **Guiding Questions** | **Probes** |
| How do you (or other stakeholders) understand, find useful and use the terms ‘health systems support’ and ‘health systems strengthening’ when designing RSSH investments?  ...also in the context of no NSP for health systems. | - Was the Global Fund RSSH guidance useful and used to help inform investment design?   - Probe on whether they find it sufficiently operational to help inform the design of investments to strengthen health systems, - How did technical assistance from RSSH experts and/or the Global Fund Country Team contribute to the design of RSSH investments? |
| What factors drive decisions on how to design RSSH investments, and how these influence the level of systems support vs systems strengthening?.  *[CEP may choose to utilize illustrative figure of where RSSH module investment is high and support or strengthening also high/low]* | Possible areas to probe further:   - Lack of involvement from HSS experts during FR/GM process - Global Fund’s role as a gap-filling mechanism, or ‘funder of last resort’ – willing to pay for activities that other donors, ministries don’t pay for (e.g. surveys, routine supervision, etc) - Emphasis on activities that are easier to implement/absorb, which tend to be more supportive - Focus on achievement of short-term targets rather than long-term sustainability   Separately, important to ask:   - How could prioritization of RSSH investments be improved? Who else needs to be at the table during the funding request? |
| **(c) Performance monitoring/measurement of RSSH:** The Global Fund modular framework was revised in 2019 to include more RSSH indicators (building on recommendations by the TRP, among others). Many countries substantially increased RSSH investments in NFM3, yet many of the new RSSH coverage indicators were not included in the NFM3 grant performance frameworks. Through this section, we are seeking to better understand how decisions are made around performance monitoring for Global Fund RSSH investments. | |
| **Guiding Questions** | **Probes** |
| How were the RSSH indicators selected for inclusion and exclusion from the Performance Framework? | - Were specific criteria used (e.g. to ensure relevance, ease of measurement, ability to measure, etc.)? - What stakeholders were involved in this process? - At what stage of the FR/GM process were these decisions taken? |
| Analysis suggests that despite investment in some RSSH modules, there are often few relevant indicators that were included in grant performance frameworks. Please could you describe why this situation arose. | - Probe on whether:   - Stakeholders were aware of new RSSH indicators in modular framework;   - The RSSH indicators were relevant and possible to measure, and felt to be important to measure; and   - The high burden of reporting already created by Global Fund monitoring requirements acted as a disincentive to introduce new indicators for RSSH. - Probe on how the performance of RSSH investments will be monitored where there are no related indicators. - What are the implications of having/not having indicators on the design of RSSH investments as supportive vs. strengthening? - Further probes on the relevance of indicators:   - Where grant activities are more focused on support, is it the case that you would not expect to make much progress against the indicators. If so, is there a disincentive to include them in the performance framework?   - Even where Global Fund support is focused on strengthening and the indicators are relevant, because they are at a high level (usually outcome) making progress against them requires a lot of other factors to come together, including the actions of stakeholders beyond the disease programs. Do stakeholders feel uncomfortable about being held accountable for progress being made against this type of RSSH indicator? |
| How do you think monitoring of RSSH investments could be improved? | - Should specific criteria / parameters be used to ensure that RSSH investments are adequately monitored? |
