# Supplementary material for: Ambitions and realities: Are Global Fund investments designed to achieve resilient and sustainable systems for health? Findings from the Global Fund Prospective Country Evaluation
Source: PLOS Glob Public Health. 2024 Nov 14;4(11):e0003914. doi: 10.1371/journal.pgph.0003914 (PMC11563383; doi:10.1371/journal.pgph.0003914)
Supplement: Attachment — Submitted filename: Rebuttal letter.docx [file pgph.0003914.s004.docx]

30 November, 2023

Dear PLOS Global Public Health Editorial Team,

This manuscript is a resubmission of manuscript PGPH-D-23-01719. The manuscript was withdrawn by the editorial team while the authors awaited clarifications to previous requests from the editorial team. We have now received those clarifications, revised our manuscript where appropriate, and have resubmitted our manuscript, as requested.

Please find below our response to the specific requests of the editorial team.

| Request from Editorial Team | Our Response |
| --- | --- |
| 1. Please report in the Methods section the day, month and year of the start and end of the recruitment period for interviews in this study. | We have added this information to the methods section. |
| 2. Also, specify in the methods section the dates when both the qualitative documents and quantitative data were accessed for research purposes. | We have added this information to the methods section. |
| 3. Please address the discrepancy within the ethics documentation. The individuals – Prof. Moses Kamya, Dr. Edgar Kestler, Monsieur Adama Faye and Trad Hatton – named as investigators on the ethics documents are not authors on the present submission. As we would generally expect the investigator named on the ethical approval to be an author on the submission, can you please provide an explanation for this situation? | This was a large, multi-year prospective evaluation involving teams of evaluators in multiple countries and two separate evaluation consortia. The ethics documentation covers the evaluation as a whole, but the principal investigators named on the ethics documentation were not directly involved in each component of the investigation, including the data collection and analysis presented in this manuscript. We have tried to be thoughtful about authorship on manuscripts, including evaluators who were involved with data collection and analysis, and to include more junior evaluators wherever appropriate. We hope this is acceptable to you. |
| 4. You mentioned that Ministry of Health approval was obtained for the EHG-led consortia. Please upload a copy of the original ethics approval letter. If the original letter is not in English, please also provide an English translated version of the supporting file. Please note that if this documentation is not included when your manuscript is resubmitted, it may be rejected. | This documentation has been uploaded with the resubmission. |

Please let us know if you require any further information.

Regards,

Nicole Salisbury
