# Supplementary material for: Ambitions and realities: Are Global Fund investments designed to achieve resilient and sustainable systems for health? Findings from the Global Fund Prospective Country Evaluation
Source: PLOS Glob Public Health. 2024 Nov 14;4(11):e0003914. doi: 10.1371/journal.pgph.0003914 (PMC11563383; doi:10.1371/journal.pgph.0003914)
Supplement: Attachment — Submitted filename: Response to Reviewers July.docx [file pgph.0003914.s005.docx]

Dear PLOS Global Public Health Editorial Team,

Many thanks for your comments. Please see below for detail on how we responded to each comment.

1. Please include a complete copy of PLOS’ questionnaire on inclusivity in global research in your revised manuscript. Our policy for research in this area aims to improve transparency in the reporting of research performed outside of researchers’ own country or community. The policy applies to researchers who have travelled to a different country to conduct research, research with Indigenous populations or their lands, and research on cultural artefacts. The questionnaire can also be requested at the journal’s discretion for any other submissions, even if these conditions are not met. Please find more information on the policy and a link to download a blank copy of the questionnaire here: <https://nam12.safelinks.protection.outlook.com/?url=https%3A%2F%2Fjournals.plos.org%2Fglobalpublichealth%2Fs%2Fbest-practices-in-research-reporting&data=05%7C02%7Cnsalisbury%40path.org%7Cba1dbfa33c8946bd1a3c08dc95e9780e%7C29ca3f4f6d6749a5a001e1db48252717%7C0%7C0%7C638550077190014470%7CUnknown%7CTWFpbGZsb3d8eyJWIjoiMC4wLjAwMDAiLCJQIjoiV2luMzIiLCJBTiI6Ik1haWwiLCJXVCI6Mn0%3D%7C0%7C%7C%7C&sdata=%2BF1VNpNUx6qbgbhM%2FO8M0emHhOGsFm3MMfgYEKmSpGg%3D&reserved=0>. Please upload a completed version of your questionnaire as Supporting Information when you resubmit your manuscript.

**Response: This statement has been completed and uploaded with our resubmission.**

2. In the ethics statement in the Methods, you have specified that verbal consent was obtained. Please provide additional details regarding how this consent was documented and witnessed, and state whether this was approved by the IRB.

**Response: we clarified how verbal consent was documented and witnessed, and that this was approved by IRB.**

3. Please provide separate main figure files in .tif or .eps format only and ensure that all files are under our size limit of 10MB. You may leave the figure captions or legends in the manuscript.

**Response from authors: saved files as .tif and uploaded with the submission.**

4. Please ensure that you refer to Figure 1 in your text as, if accepted, production will need this reference to link the reader to the figure.

**Response from authors: added reference to Figure 1 in text.**

5. Please amend your detailed online Financial Disclosure statement. This is published with the article. It must therefore be completed in full sentences and contain the exact wording you wish to be published.

a) State the initials, alongside each funding source, of each author to receive each grant. For example: "This work was supported by the National Institutes of Health (####### to AM; ###### to CJ) and the National Science Foundation (###### to AM)."

**Response from authors: all authors on this manuscript received funding from the Global Fund to conduct this work through the Prospective Country Evaluation grant. We have clarified this in the financial disclosure statement.**

b) State what role the funders took in the study. If the funders had no role in your study, please state: “The funders had no role in study design, data collection and analysis, decision to publish, or preparation of the manuscript.”

**Response: we have updated to specify that the funder, in this case the Technical Evaluation Reference Group reviewed the work, though they did not request that we make substantive edits.**

6. Please update your online Competing Interests statement. If you have no competing interests to declare, please simply state: “The authors have declared that no competing interests exist.”

**Response from authors: We have updated this statement per your suggestion.**

7. We note your current Data Availability statement is:

"Quantitative analyses were based on third-party data (detailed grant budgets) accessed directly from the Global Fund and the authors are not legally able to distribute this data set. While detailed budgets are not presently publicly available on the Global Fund's website, interested parties could seek access through contacting the Global Fund and/or Principal Recipients of the Global Fund grants. The qualitative data transcripts that support the findings cannot be shared publicly per the terms under which respondents were consented to participate. However, analysis tables may be available upon reasonable request to the authors and with the permission of all organizations within the research consortia and permission of the Global Fund."

As this are third party data that cannot be shared publicly by the authors, please provide non-author contact information for the Global Fund to which data requests may be sent.

Please update your Data Availability statement to include the information you provide.

**Response from authors: we have updated the data availability statement and directed interested parties to the Global Fund Independent Evaluation Panel, which has replaced the Technical Evaluation Reference Group that originally commissioned this evaluation.**
